# Impact of Contact Force-Sensing Catheters on Fluoroscopy Time in Interventional Electrophysiology: A European Survey

**DOI:** 10.3390/jcm11051322

**Published:** 2022-02-28

**Authors:** Lukas Fiedler, Hermann Blessberger, Pawel Balsam, Tom De Potter, Piotr Buchta, Sabine Ernst, Victor Waldmann, Francisco Moscoso Costa, Stefan Bogdan, Alexander Nahler, Denis Hrncic, Thomas Lambert, Robert Schönbauer, Michael Pfeffer, Franz Xaver Roithinger, Clemens Steinwender, Jedrzej Kosiuk

**Affiliations:** 1 Department of Internal Medicine, Cardiology, Nephrology and Intensive Care Medicine, Hospital Wiener Neustadt, 2700 Wiener Neustadt, Austria; lukas.fiedler@wienerneustadt.lknoe.at (L.F.); michael.pfeffer@wienerneustadt.lknoe.at (M.P.); franzxaver.roithinger@wienerneustadt.lknoe.at (F.X.R.); 2 Department of Internal Medicine II, Paracelsus Medical University, 5020 Salzburg, Austria; 3 Department of Cardiology, Kepler University Hospital, Medical Faculty, Johannes Kepler University Linz, Krankenhausstrasse 9, 4021 Linz, Austria; alexander.nahler@kepleruniklinikum.at (A.N.); denis.hrncic@kepleruniklinikum.at (D.H.); thomas.lambert@kepleruniklinikum.at (T.L.); clemens.steinwender@kepleruniklinikum.at (C.S.); 4 First Department of Cardiology, Warsaw Medical University, 02-097 Warsaw, Poland; pawel.balsam@wum.edu.pl; 5 Department of Cardiology, OLV Hospital, 9300 Aalst, Belgium; tom.de.potter@olvz-aalst.be; 6 Silesian Center for Heart Diseases, 41-800 Zabrze, Poland; piotr.buchta@gmail.com; 7 Department of Cardiology, Royal Brompton & Harefield Hospital, Imperial College London, London SW7 2BX, UK; s.ernst@rbht.nhs.uk; 8 Department of Cardiology, Georges Pompidou European Hospital, 75015 Paris, France; victor.waldmann@gmail.com; 9 Department of Cardiology, Hospital de Santa Cruz, 2790-134 Carnaxide, Portugal; fmoscosocosta@gmail.com; 10 Department of Cardiology, Clinical Emergency Hospital, Carol Davila University of Medicine and Pharmacy, 014461 Bucharest, Romania; bogdan.stefan@umfcd.ro; 11 Department of Cardiology, Medical University of Vienna, 1090 Vienna, Austria; robert.schoenbauer@meduniwien.ac.at; 12 Department of Cardiology, Helios Klinik Koethen, 06366 Koethen, Germany; jedrzej.kosiuk@helios-gesundheit.de

**Keywords:** current practice, electrophysiology, catheter ablation of arrhythmias, fluoroscopy, contact force-sensing catheters

## Abstract

This multicenter European survey systematically evaluated the impact of using contact force-sensing catheters (CFSCs) on fluoroscopy and procedure time in interventional electrophysiology. Data from 25 participating centers were collected and analyzed, also considering important confounders. With the use of CFSCs, fluoroscopy time was reduced for right- and left-sided atrial ablations (median −6.4 to −9.6 min, *p* < 0.001 for both groups), whereas no such effect could be found for ventricular ablations. Moreover, the use of CFSCs was associated with an increase in procedure time for right-sided atrial and ventricular ablations (median +26.0 and +44.0 min, respectively, *p* < 0.001 for both groups), but not for left-sided atrial ablations. These findings were confirmed independent of career level and operator volume, except for very highly experienced electrophysiologists, in whom the effect was blunted. In the subset of pulmonary vein isolations (PVIs), CFSCs were shown to reduce both fluoroscopy and procedure time. In conclusion, the use of CFSCs was associated with a reduced fluoroscopy time for atrial ablations and an increased procedure time for right atrial and ventricular ablations. These effects were virtually independent of the operator experience and caseload. When considering only PVIs as an important subset, CFSCs were shown to reduce both fluoroscopy and procedure time.

## 1. Introduction

Contact force-sensing catheters (CFSCs) provide instantaneous feedback regarding the tissue catheter interface, thus making the ablation lesions more predictable. For pulmonary vein isolations (PVIs), this technology has been shown to be associated with a significant reduction in atrial fibrillation recurrences and an equivalent safety profile as compared with conventional ablation catheters [1]. However, the impact of this technology on procedure and fluoroscopy time is less certain, and meta-analyses have yielded conflicting results [2,3,4]. As patients accumulate radiation doses over a lifetime, it is crucial to restrict the use of fluoroscopy to a minimum according to the ALARA (as low as reasonably achievable) principle [5,6]. Besides patients, operators and other medical personnel, such as nurses and technicians, are also regularly exposed to radiation. For professionals working in cardiac catheterization laboratories, the additional attributable lifetime risk for fatal or non-fatal cancer has been shown to be approximately 1 in 200 [7]. Likewise, the additional attributable lifetime risk of developing a malignancy was calculated to be 0.16% if a patient had undergone a single pulmonary vein isolation with a median fluoroscopy time of 23 min [8]. This seems particularly noteworthy, as the mean fluoroscopy time for pulmonary vein isolations is longer than in electrophysiological (EP) procedures for the treatment of other supraventricular arrhythmias [9]. Due to the demographic shift, the number of patients undergoing pulmonary vein isolations is on a steep rise. The use of CFSCs for pulmonary vein isolations and other EP procedures may be potentially beneficial to both patients and staff by reducing fluoroscopy time. With this European survey, we sought to investigate the contemporary use of CFSCs and their impact on fluoroscopy and procedure time.

## 2. Materials and Methods

### 2.1. Study Design and Main Objective

This study was a European multicenter observational survey of consecutive EP procedures of any kind. European EP centers were asked to participate in the project. The study was conducted in accordance with the Declaration of Helsinki and local ethical standards depending on the regulations in each country. All electrophysiological procedures were performed according to the institutions’ standard operating procedures. The use of a 3D mapping system or a contact force-sensing ablation catheter was at the operators’ discretion.

### 2.2. Data Collection and Reporting

The participating centers were asked to complete a pre-defined, structured questionnaire that comprised information about the center and the operators as well as anonymized details of the electrophysiological procedures. An emphasis was put on center and operator experience (number of procedures per center and per operator and the career level of operators) as well as procedural data (exact type of procedure, fluoroscopy time, procedure time, use of a 3D mapping system, type of ablation catheter with or without contact force sensing, and body mass index (BMI) of the patient). To avoid a potential bias caused by the predominance of data from high-volume centers, the number of operators per center was limited to five, with a maximum of 20 most recent consecutive exams for each of them. Data were reported according to the STROBE statement for cross-sectional studies [10].

### 2.3. Statistical Analysis

Data from electrophysiological procedures that were diagnostic only, AV nodal ablations (mandatory use of fluoroscopy to visualize pacemaker leads), or procedures that applied single shot devices for pulmonary vein isolation were excluded from the analysis. To enhance the sample size and statistical power, we grouped the EP exams into left-atrial, right-atrial, and ventricular EP procedures for the main analysis. As operator experience and operator caseload were likely to affect fluoroscopy usage and procedure time to a considerable extent, we also analyzed the data with respect to these potential confounders. In addition, the data were also analyzed with a focus on further confounders, such as center volume, BMI, the use of a 3D mapping system, and the exact type of EP procedure. Stratifications for all these confounders (sensitivity analyses) were performed to check for the impact of these parameters on the overall results. The standard distribution of continuous variables was visually and formally assessed (Shapiro–Wilk test). Median values with interquartile ranges (ranging between the 25th and 75th percentile) or mean values with standard deviations were reported, as appropriate. For categorical data, absolute numbers and percentages were presented. As the Student’s *t*-test has been proven to be robust for large sample sizes, even with skewed data, it was applied to compare continuous variables with a Welch modification for unequal variances, when indicated [11]. Categorical variables were compared using the χ^2^-test. All calculations were performed with the software package Intercooled Stata (Version 14, Stata Corp., College Station, TX, USA).

## 3. Results

### 3.1. Baseline Characteristics of Participating Centers and Operators

Twenty-five centers from 14 European countries participated in the survey (Table S1). Most EP labs were affiliated with a university hospital (18 centers, 72%), whereas two centers (8%) were in a non-academic tertiary care setting and five (20%) were in a public secondary care setting. The number of ablation procedures per center varied between 130 and 1600 per year (median: 420, interquartile range (IQR): 285–500) performed by one to seven electrophysiologists per EP center (median: 5, IQR: 4–7). The participating electrophysiologists (*n* = 98) were between 29 and 65 years old (median 39, IQR: 36–44) and predominantly male (*n* = 84, 85.7%). All levels of operator experience were well represented (early career, <5 years of practical experience: 32 (32.7%); mid-career, between 5 and 15 years: 43 (43.9%); mentor status, >15 years: 23 (23.5%)). Most electrophysiologists performed less than 40 procedures per month (1–9 procedures: 29 (29.6%); 10–19: 27 (27.6%); 20–39: 21 (21.4%); >40: 17 (17.3%); no comment: 4 (4.1%)).

### 3.2. Types of Procedures and Use of 3D Mapping Systems

Overall, data were collected from 1788 EP procedures, of which 1425 (79.9%) were eligible for our analysis. The exclusion of 363 EP studies was due to the following reasons: single shot device for PVI (*n* = 126, 7.0%), diagnostic examinations only (*n* = 154, 8.6%), and AV nodal ablations (*n* = 66, 3.7%). EP studies conducted for the treatment of complex scar-related supraventricular tachyarrhythmias (*n =* 17, 1.0%) were also excluded, as they were deemed not representative of a standard EP examination and could not be further classified as right or left atrial procedures. The remaining 1425 ablations were divided into three groups: (1) right-sided atrial procedures (*n* = 646, 45.3%, group 1); (2) left-sided atrial procedures (*n* = 622, 43.6%, group 2); and (3) ventricular procedures (*n* = 157, 11.0%, group 3), such as the ablation of ventricular tachycardias (VTs) and premature ventricular contractions (PVCs). When looking at ventricular tachycardia ablations in detail, a sole antegrade access was used for *n* = 33 VT ablations (50%), a sole retrograde access for *n* = 6 VT ablations (9.1%), and a combined access (antegrade and retrograde (*n* = 25) or antegrade and epicardial/subxiphoidal (*n* = 2)) for *n* = 27 VT ablations (40.9%). Table 1 gives a detailed overview of the number and peri-procedural parameters of different arrhythmias. A 3D mapping system was employed in about two thirds of the EP studies (*n* = 961, 67.4%: Biosense Webster Carto 3^®^ *n* = 543 (56.5%), Abbott EnSite NavX^®^ *n* = 296 (30.8%), Boston Scientific Rhythmia^®^ *n* = 33 (3.4%), and other systems *n* = 89 (9.3%)). Image fusion (NavX Mediguide^®^ *n* = 21 (1.5%) and Carto Univu^®^ *n* = 162 (11.4%)), magnetic remote navigation (*n* = 7 (0.5%)), or robotic navigation (Hansen^®^ *n* = 11 (0.8%)) were used infrequently. No EP lab routinely applied intracardiac ultrasound. Of all EP procedures analyzed, 99 (6.9%) were performed solely guided by a 3D mapping system, and without the use of fluoroscopy (truly “zero fluoro” in the strict sense, with a dose area product (DAP) of 0.0 cGy* cm^2^). This “zero fluoro” strategy was applied in 80 right-sided atrial procedures (34 of which used CFSCs), nine left-sided atrial procedures (seven of which used CFSCs), and in 10 ventricular ablation procedures (nine of which used CFSCs). Right atrial “zero fluoro” procedures comprised 26 slow pathway ablations, 44 cavo-tricuspid isthmus ablations, eight accessory pathway ablations, and the ablation of two atrial tachycardias. The group of left atrial “zero fluoro” procedures included six pulmonary vein isolations and three accessory pathway ablations, whereas nine ablations of ventricular extra beats and one VT ablation were attributed to the ventricular group. The body mass index (BMI) of patients undergoing an EP procedure was 27.5 ± 5.2 kg/m^2^ on average (BMI data from 66 (4.6%) examinations were missing).

### 3.3. Main Analysis: Impact of Contact Force-Sensing Catheters (CFSCs) on Fluoroscopy and Procedure Time According to EP Procedure Group

**Figure 1:**
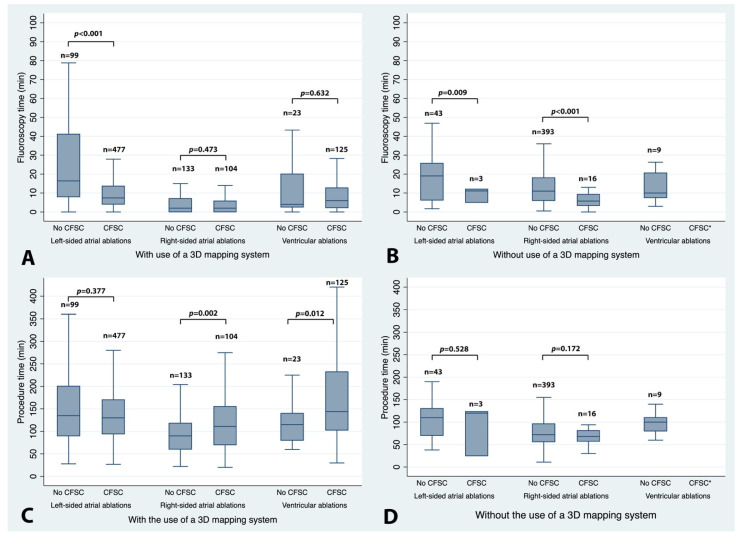
Impact of CFSCs on fluoroscopy and procedure time according to the type of EP procedure and the use of a 3D mapping system. Box and whisker plots depicting the impact of contact force-sensing catheters (CFSCs) on fluoroscopy time (upper panels (**A**,**B**)) and procedure time (lower panels (**C**,**D**)) according to the type of EP procedure with (left panels (**A**,**C**)) and without (right panels (**B**,**D**)) the use of a 3D mapping system. Boxes represent 25th and 75th percentiles with the median as a solid center line. Whiskers indicate the most extreme values within 1.5 times the interquartile range above the 75th percentile and below the 25th percentile. Outside values were not plotted. * No observations. No ventricular ablations were performed with CFSCs but without 3D mapping.

Fluoroscopy time was significantly reduced with the use of CFSCs in left- and right-sided atrial EP procedures, while we could not detect an effect for the ablation of ventricular arrhythmias (Table 2, Figure 1). The use of CFSCs was associated with longer procedure times for right-atrial and ventricular ablations, but not for left-atrial procedures.

### 3.4. Impact of Career Level, Operator Caseload, Center Volume, and Body Mass Index

Complete data on the level of operator experience and center volume were available. However, for 50 (3.5%) examinations, the number of EP procedures performed by the operator per month was missing, and BMI data were missing for 66 exams (4.6%). When stratifying for operator experience and volume, the usage of CFSCs shortened fluoroscopy times throughout all career levels and operator volumes, except for high-volume operators performing more than 40 procedures per month (Table 3, 3A and 3B, Figure S1). As previously seen in the main analysis, CFSCs led to consistently longer procedure times in all these strata, except for operators at the mentor level. When center volume was studied in detail, the positive effect of CFSCs on fluoroscopy time was blunted, except for centers performing 500–999 procedures per year (Table 3, 3C). However, a significant lengthening of procedures with the use of CFSCs could still be detected in all four strata. Finally, the reduction in fluoroscopy time by CFSCs was confirmed for all three defined BMI levels (Table 3, 3D). Concordantly, the procedure time was increased for all BMI strata, except for a BMI below 20 kg/m^2^. Compared to the other two BMI groups, the stratum with a BMI below 20 kg/m^2^ was small and comprised only 64 EP studies.

### 3.5. Impact of EP Procedure Type and Use of 3D Mapping Systems

The data on the exact type of procedure as well as the use of a 3D mapping system were collected for every EP examination (Figure 1, Table S2A). Most right-sided atrial EP procedures were performed without the use of both a 3D mapping system and a CFSC (393 out of 646 (60.8%)). Right atrial tachycardias, which can have a more complex underlying mechanism and may be technically more demanding, were the only exception to the rule and were more often ablated with 3D mapping systems and CFSCs (20 out of 38 (52.6%)). Conversely, in left-sided EP procedures, 3D mapping systems with CFSCs were frequently used (576 out of 622 (92.6%)), except for accessory pathways that were mainly ablated without the use of either of these. Similarly, in most ventricular EP ablations, both a 3D mapping system as well as a CFSC were used (148 out of 157 (94.3%)). When looking at the data in detail, the overall reduction in fluoroscopy time in right-sided atrial EP procedures was mainly driven by using CFSCs for slow pathway ablations. This effect was found with or without 3D mapping (Table S2B). Conversely, the overall prolongation of the procedure time was mainly caused by a longer procedure duration in flutter ablations using both CFSCs and a 3D mapping system (median 70 min vs. 118 min, Table S2C, Figure S1). In group 2 (left-sided atrial EP procedures), the overall fluoroscopy time was primarily shortened by pulmonary vein isolations that used a CFSC and a 3D mapping system (median reduction of 11.8 min for PVI only and 38.0 min for PVI with additional lesions). The procedure time was shorter by a median of 50 min if all the PVIs were pooled. This finding was less pronounced if only PVIs without additional lesions were analyzed (median reduction of 12 min for PVIs only vs. 57 min for PVIs with additional lesions). If all of the group 2 left atrial procedures were considered, no change in procedure time could be detected. Obviously, the shorter procedure times of PVIs performed with CFSCs were offset by the longer procedure times of left accessory pathway or atrial tachycardia ablations performed with CFSCs with or without 3D mapping. For ventricular ablations (group 3), no overall effect on the fluoroscopy time could be found, whereas the overall prolongation of the procedure time was mainly caused by PVC ablations that applied a 3D mapping system and a CFSC.

## 4. Discussion

In summary, when analyzing the data from this observational cross-sectional European survey, we were able to identify a certain pattern, namely that the use of CFSCs was associated with a shortening of fluoroscopy time (median reduction between 6.4 and 9.6 min in right- and left-sided procedures, respectively) and a prolongation of procedure time (median increase of 26 and 44 min in right-sided and ventricular procedures, respectively). We could not identify an impact of CFSCs on fluoroscopy time in ventricular procedures and on procedure time in left-sided atrial ablations. These phenomena most likely reflect the fact that operators instantaneously try to improve catheter positioning based on the feedback provided by the CFSC [12]. This action takes time, but usually does not require the use of fluoroscopy, especially when working with a 3D mapping system that allows for fluoro-less catheter visualization. A more precise and effective placing of ablation points with CFSCs has been shown to translate into better clinical results, e.g., after pulmonary vein isolations performed for the treatment of atrial fibrillation [2,3].

### 4.1. Impact of Career Level, Operator Caseload, Center Volume, and BMI

In general, the pattern outlined above could be demonstrated uniformly for all career levels and all stages of operator experience with just two exceptions: fluoroscopy time was not significantly shortened and procedure time was not significantly prolonged in very experienced operators (performing either more than 40 EP procedures per month or having more than 15 years working experience). This finding can most likely be explained by their high caseload over many years and their motor skills that have been extensively trained as a result. In such experienced hands, the advantages and shortcomings of CFSCs seem to vanish, whereas the vast majority of electrophysiologists may benefit from CFSCs in terms of fluoroscopy time reduction and safety [13]. When the data were analyzed according to center volume, the findings were more inconsistent. Whereas longer procedure times were detected in all strata, a pronounced reduction in fluoroscopy time was only found in centers with 500–999 EP cases per year, which contributed about 22% of all EP cases. This may reflect a different range of procedures at centers of different sizes. For example, more ventricular ablations without a substantial reduction in fluoroscopy time are likely to be performed at large centers, and pulmonary vein isolations are mainly performed at medium to large centers. When considering the increase in potentially hazardous scattered radiation with a higher patient BMI, it is encouraging to see that fluoroscopy times were shortened in all BMI groups, whereas the procedure time was prolonged at a BMI level above 20 kg/m^2^ only. However, the data derived from the lowest BMI group (<20 kg/m^2^) must be interpreted cautiously, as this group was small and comprised only 64 EP procedures.

### 4.2. Right-Sided Atrial Procedures

Sub-analyses revealed that the shortening of the fluoroscopy time in this group was mainly driven by slow pathway ablations and cavo-tricuspid isthmus ablations without the use of 3D mapping systems. Obviously, the contact force information helped with navigation within the right atrium, thereby saving fluoroscopy time. However, fluoroscopy time was also shortened in slow pathway ablations if a 3D mapping system was applied. Hence, it can be hypothesized that contact force information may help in validating the 3D anatomical model and navigating in it, thus saving fluoroscopy time. The prolongation of procedure time in this group was mainly caused by cavo-tricuspid isthmus ablations applying both CFSCs and 3D mapping systems, whereas no such effect was found for CFSCs without the use of a 3D mapping system. The following points may have contributed to this finding: the generation of the 3D anatomy, frequent repositioning to achieve optimal contact force, and remaining longer on the ablation point to generate optimal ablation lesions (as reflected by an adequate force–time integral, lesion size index, or ablation index) take time. In addition, studies with different waiting strategies after reaching the ablation end point (bidirectional isthmus block) may have been randomly grouped within one of the two catheter cohorts. Our results are in contrast with a randomized controlled trial comprising 156 participants [14]. The study compared atrial flutter ablations using a 3D mapping system with or without measuring contact force. No differences in fluoroscopy and procedure times could be detected. The average fluoroscopy time in this study was longer (median 8 min vs. 2 min), and the procedure time without a scheduled waiting period was shorter (median 50 min vs. 118 min) than in our study.

### 4.3. Left-Sided Atrial Procedures

For left atrial ablations, fluoroscopy time was reduced overall with the use of CFSCs, whereas no effect was observed on procedure time. However, sub-analyses focusing on pulmonary vein isolations applying a 3D mapping system—representing the largest sub-group—revealed that both fluoroscopy and procedure time were shortened. The positive effect on procedure time was offset after pooling the pulmonary vein isolations with the other left atrial ablations. CFSCs likely perform best if used for highly standardized pulmonary vein isolations because they facilitate the validation of the 3D map and navigation within it, and enable more effective ablation points, rendering the search for gaps unnecessary in many cases. Our findings are in line with a meta-analysis that found a reduction in both fluoroscopy and procedure time with the use of CFSCs [3]. This study scrutinized data retrieved from eleven trials of patients undergoing pulmonary vein isolations, of which two were randomized controlled trials. Pooled data from 1428 patients revealed that the average procedure time was shortened by 17 min and the average fluoroscopy time by 8 min. The authors hypothesized that less visual guidance was necessary with CFSCs, leading to the cut in fluoroscopy times, whereas more effective lesions with CFSCs enabled faster pulmonary vein isolations without the need for remapping and closing gaps caused by residual or dormant conduction [3,12]. Both the reduction in fluoroscopy and procedure times with the use of CFSCs were more pronounced in our study (median reduction in fluoroscopy and procedure time 11.8–38.0 min and 12.0–57.0 min, respectively), maybe due to different institutional protocols for conducting PVIs and the different equipment used. As opposed to these findings, another meta-analysis analyzing data from pulmonary vein isolations found no reduction in fluoroscopy and procedure times with the use of CFSCs [2]. In addition to the use of CFSCs, a recent meta-analysis of 11 studies involving 2306 patients found the use of the ablation index (AI) as a standardized protocol for pulmonary vein isolation to be beneficial [15]. The AI integrates contact force, time, and power settings into an index reflecting lesion size and effectiveness. This meta-analysis showed that the use of the AI resulted in significantly shorter procedure and fluoroscopy times (on average −11.8 min and −1.8 min, respectively), even when a proportion of patients in the control groups were also treated with CFSCs but without the standardized ablation protocol. This effect may also have contributed to the shorter fluoroscopy and procedure times observed in the subgroup of pulmonary vein isolations in our survey.

### 4.4. Ventricular Procedures

While the use of CFSCs was associated with an overall longer procedure time, which was mainly driven by the ablations of PVCs, no effect was found regarding fluoroscopy time. The small number of ventricular ablation procedures, the lack of standardization—especially of VT ablations—in this still evolving field, the heterogeneity of underlying myocardial pathologies, and the complexity of these ablations may explain the finding that a certain amount of fluoroscopy was still necessary despite the use of CFSCs. Frequent repositioning of the ablation catheter in the ventricle with its trabeculated endocardial surface to achieve an adequate contact force may have contributed to the prolonged procedure time for ventricular ablations with CFSCs. Our findings contrast with two recent retrospective studies that investigated the impact of CFSCs on the ablation of ventricular outflow tract PVCs [16,17]. These trials reported no differences in fluoroscopy or procedure time with the use of CFSCs.

### 4.5. Limitations

This study was a non-randomized observational survey by design, with all the limitations inherent in such. We cannot rule out a reporting bias due to self-reporting with a structured questionnaire. Furthermore, it is possible that more complex EP cases were more likely to be conducted with CFSCs, which may have introduced a selection bias. In addition, different centers and operators may have used different standard operating procedures for ablations (e.g., using standardized X-ray projections or applying waiting times) that led to a reduction or an increase in fluoroscopy and/or procedure time. Fluoroscopy time is only a proxy parameter for radiation dose as assessed by the dose area product. These two parameters are not interchangeable, as the DAP is—for instance—also influenced by the BMI of the patient; the generation of the X-ray system; and machine settings, such as filtering. However, fluoroscopy time is the most important parameter that can be influenced by the electrophysiologist. Procedural success rates, periprocedural complications, long-term follow-up data, and the exact types and brand names of ablation catheters used were not collected, precluding further analysis. For the ablation of ventricular tachycardias, the exact location of the ablation (right ventricle, left ventricle, both ventricles, or exact location of scar) as well as the type of cardiomyopathy and indication for ablation were not recorded because this was beyond the scope of this survey.

## 5. Conclusions

In our observational European survey, we demonstrated that CFSCs helped to reduce fluoroscopy time in right- or left-sided atrial ablation procedures, whereas no effect was found in ventricular ablations. The procedure time was increased with the use of CFSCs in right-sided atrial procedures and ventricular ablations, but not in left-sided atrial ablations. Shorter fluoroscopy times and longer procedure times were virtually independent of operator experience and caseload. When looking only at PVIs as an important sub-group, CFSCs were shown to reduce both fluoroscopy and procedure time, which is of major clinical relevance in everyday practice.

## Figures and Tables

**Table 1:** Number of different types of procedures with peri-procedural details.

Type of Arrhythmia/Procedure	Overall Number of Studies	Number of EP Studies Using a CFSC	Number of EP Studies Using 3D Mapping	Number of EP Studies Using a CFSC + 3D Mapping	Median Fluoroscopy Time (min) *	Median Procedure Time (min) *
AV nodal reentrant tachycardia (AVNRT)	267	27 (10.1%)	70 (26.2%)	19 (7.1%)	8.0 (3.0–13.3) [0.0–48.0]	78.0 (60.0–110.0) [13.0–259.0]
Cavo-tricuspid isthmus ablation	301	63 (20.9%)	121 (40.2%)	56 (18.6%)	6.0 (2.0–15.0) [0.0–48.1]	70.0 (55.0–103.0) [11.0–275.0]
Right atrial tachycardia	38	20 (52.6%)	27 (71.1%)	20 (52.6%)	7.1 (4.0–20.0) [0.0–55.6]	120.0 (90.0–140.0) [29.0–307.0]
Right-sided accessory pathway ablation	40	10 (25.0%)	19 (47.5%)	9 (22.5%)	8.5 (2.5–19.6) [0.0–60.3]	90.0 (60.0–155.0) [28.0–240.0]
Pulmonary vein isolation (RF)	393	352 (89.6%)	393 (100.0%)	352 (89.6%)	8.0 (4.2–14.0) [0.0–70.4]	124.0 (92.0–167.0) [27.0–407.0]
Pulmonary vein isolation with additional lesions (RF)	122	95 (77.9%)	122 (100.0%)	95 (77.9%)	10.0 (5.0–20.0) [0.0–119.6]	169.5 (130.0–210.0) [36.0–425.0]
Left atrial tachycardia	42	23 (54.8%)	35 (83.3%)	22 (52.4%)	9.0 (6.0–20.4) [0.0–128.1]	114.5 (60.0–180.0) [25.0–543.0]
Left-sided accessory pathway ablation	65	10 (15.4%)	26 (40.0%)	8 (12.3%)	8.5 (4.0–21.0) [0.0–84.6]	94.5 (65.0–121.5) [28.0–270.0]
VT ablation	66	56 (84.9%)	66 (100.0%)	56 (84.8%)	9.9 (4.0–23.0) [0.0–73.0]	180.0 (123.0–260.0) [46.0–420.0]
PVC ablation	91	69 (75.8%)	82 (90.1%)	69 (75.8%)	5.0 (2.0–8.0) [0.0–33.1]	110.5 (80.0–159.0) [30.0–360.0]
Overall	1425	725 (50.9%)	961 (67.4%)	706 (49.5%)	8.0 (3.3–15.2) [0.0–128.1]	105.0 (70.0–150.0) [11.0–543.0]

CFSC—contact force-sensing ablation catheter; RF—radiofrequency energy; AV—atrioventricular; VT—ventricular tachycardia; PVC—premature ventricular contraction. * Median values with interquartile ranges (round brackets) and minimum to maximum ranges (squared brackets).

**Table 2:** Main analysis. Fluoroscopy and procedure times per group with and without the use of contact force-sensing ablation catheters.

Group	Number of EP Studies (Overall/w/o CFSC/w CFSC) *	Fluoro Time w/o CFSC (min) ^†^	Fluoro Time w CFSC (min) ^†^	*p*-Value ^‡^	Procedure Time w/o CFSC (min) ^†^	Procedure Time w CFSC (min) ^†^	*p*-Value ^‡^
Group 1 (right-sided atrial procedures)	646/526/120	9.0 (4.0–16.0)	2.6 (0.0–6.0)	<0.001	75.0 (57.0–105.0)	101.0 (65.0–150.0)	<0.001
Group 2 (left-sided atrial procedures)	622/142/480	17.0 (7.0–38.0)	7.4 (4.0–13.2)	<0.001	124.0 (85.0–180.0)	130.0 (93.5–170.0)	0.721
Group 3 (ventricular ablation procedures)	157/32/125	7.0 (3.0–20.3)	6.0 (2.2–12.7)	0.424	100.0 (80.0–127.5)	144.0 (102.5–232)	<0.001

Numbers are presented for different groups of EP procedures. * Absolute numbers. ^†^ Data are presented as medians with interquartile ranges. ^‡^ Student’s *t*-test with Welch modification. w—with; w/o—without; CFSC—contact force-sensing catheter.

**Table 3:** A–D. Fluoroscopy and procedure times with and without the use of CFSCs for different confounders.

Group	Number of EP Studies (Overall/w/o CFSC/w CFSC) *	Fluoro Time w/o CFSC (min) ^†^	Fluoro Time w CFSC (min) ^†^	*p*-Value ^‡^	Procedure Time w/o CFSC (min) ^†^	Procedure Time w CFSC (min) ^†^	*p*-Value ^‡^
3A: Operator career level							
Early career	421/247/174	10.5 (6.0–19.7)	7.0 (3.0-13.0)	<0.001	75.0 (60.0–113.0)	140.0 (105.0–180.0)	<0.001
Mid-career	620/306/314	7.5 (2.7–18.0)	6.0 (3.0-9.5)	<0.001	83.0 (60.0–120.0)	130.0 (94.0–180.0)	<0.001
Mentor	384/147/237	12.0 (6.0–19.0)	7.5 (2.6–15.0)	<0.001	100.0 (60.0–140.0)	119.0 (83.0–155.5)	0.108
3B: Operator caseload per month							
1–9/mo	362/223/139	12.0 (6.0–25.0)	6.0 (1.3–14.0)	<0.001	90.0 (64.0–127.0)	130.0 (90.0–180.0)	<0.001
10–19/mo	380/177/203	7.0 (0.8–18.0)	6.0 (3.2–10.1)	<0.001	96.0 (70.0–135.0)	150.0 (105.0–180.0)	<0.001
20–39/mo	337/167/170	11.0 (6.0–18.0)	7.5 (3.2–13.0)	<0.001	70.0 (40.0–110.0)	120.0 (85.0–169.0)	<0.001
>40/mo	296/103/193	8.0 (4.0–14.0)	6.1 (3.0–13.0)	0.406	70.0 (55.0–96.0)	120.0 (90.0–165.0)	<0.001
3C: Cases per center per year							
<200/yr	36/17/19	4.0 (0.0–22.0)	5.0 (0.0–7.0)	0.197	120.0 (102.5–145.0)	190.0 (150.0–215.0)	<0.001
200–499/yr	767/418/349	9.0 (4.0–15.0)	7.5 (4.0–14.9)	0.959	83.0 (60.0–115.0)	150.0 (120.0–185.0)	<0.001
500–999/yr	311/191/120	17.0 (8.0–35.6)	9.0 (5.0–14.0)	<0.001	90.0 (60.0–140.0)	120.0 (87.5–182.5)	<0.001
≥1000/yr	311/74/237	4.0 (2.1–11.0)	4.2 (2.0–8.0)	0.286	80.0 (60.0–113.0)	97.0 (76.0–129.0)	0.018
3D: Patient BMI level							
BMI <20	64/34/30	10.0 (4.0–14.3)	3.8 (1.0–11.1)	0.023	113.0 (70.0–130.0)	98.0 (60.0–165.0)	0.876
BMI 20–30	914/476/438	10.0 (4.0–18.6)	6.9 (3.0–13.0)	<0.001	83.0 (60.0–120.0)	124.0 (90.0–180.0)	<0.001
BMI >30	381/169/212	10.0 (4.0–20.0)	7.3 (3.1–13.0)	<0.001	80.0 (59.0–130.0)	129.0 (95.0–170.0)	<0.001

Numbers are presented for different career levels (3A), the number of EP procedures per operator per month (3B), center volumes (3C, procedures per year), and the BMI levels (body mass index in kg/m^2^) of treated individuals (3D). * Absolute numbers. ^†^ Data are presented as medians with interquartile ranges. ^‡^ Student’s *t*-test with Welch modification. w—with; w/o—without; CFSC—contact force-sensing catheter.

## Data Availability

The data presented in this study are available on request. The data are not publicly available due to privacy reasons, as well as provision from third parties.

## Supplementary Materials

The following are available online at https://www.mdpi.com/article/10.3390/jcm11051322/s1, Figure S1: Impact of CFSCs on fluoroscopy and procedure time with respect to major confounders, Table S1: Details of contributing countries and centers, Table S2A: Overview of the number of different EP procedures per group and the respective use of a 3D mapping system and/or contact force-sensing catheters, Table S2B: Fluoroscopy times with and without the use of contact force-sensing ablation catheters for different EP procedures and according to the use of a 3D mapping system, Table S2C: Procedure times with and without the use of contact force-sensing ablation catheters for different EP procedures and according to the use of a 3D mapping system.
