# Supplementary material for: Impact of Contact Force-Sensing Catheters on Fluoroscopy Time in Interventional Electrophysiology: A European Survey
Source: J Clin Med. 2022 Feb 28;11(5):1322. doi: 10.3390/jcm11051322 (PMC8911176; doi:10.3390/jcm11051322)
Supplement: Supplementary file 1 [file jcm-11-01322-s001.zip › jcm-1607049-supplementary.pdf]

Supplementary Materials

Figure S1. Impact of CFSCs on fluoroscopy and procedure time with respect to major confounders.

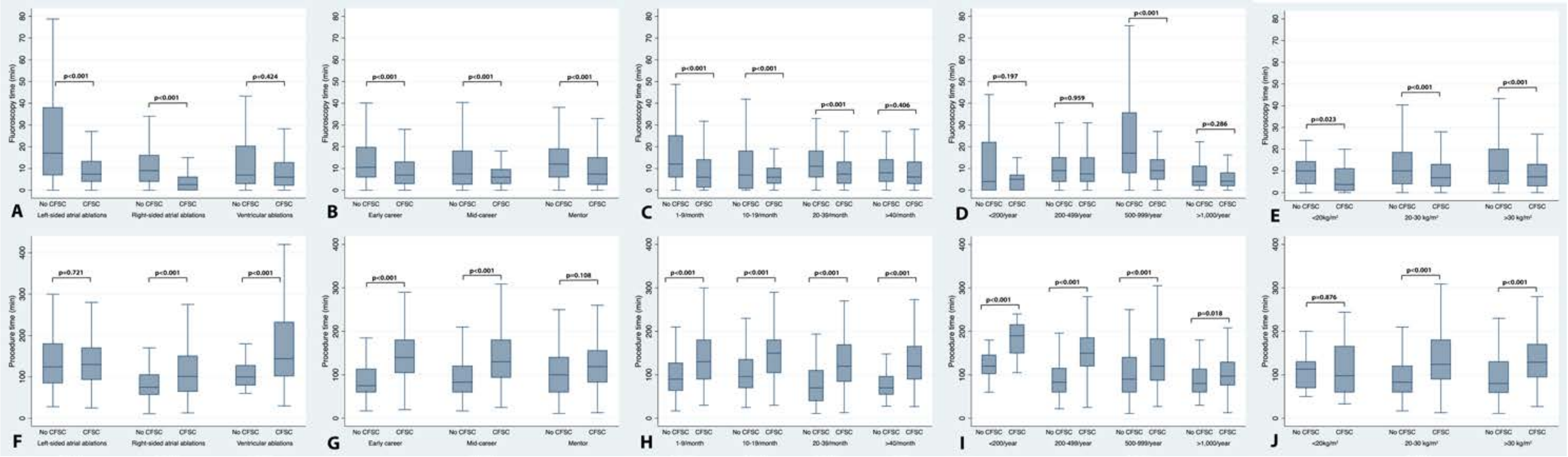

Figure S1. Box and whiskers plots depicting the impact of contact force sensing catheters (CFSCs) on fluoroscopy time (upper panels A-E) and procedure time (lower panels F-J) according to type of EP procedure (panels A+F), level of operator experience (panels B+G), operator volume (procedures per month, panels C+H), center volume (ablations per year, panels D+I) as well as the patients' body mass index (BMI, panels E+J). Boxes represent 25th and 75th percentiles with the median as a solid center line. Whiskers indicate the most extreme values within 1.5 times the interquartile range above the 75th percentile and below the 25th percentile, respectively. Outside values were not plotted.

Table S1. Details of contributing countries and centers.

Country	Number of centers	University hospitals	Tertiary care centers	Public hospitals	Overall number of operators	Overall number of procedures	Number of procedures included in this analysis
**Austria**	2	1	0	1	10	187	153
**Belgium**	1	0	1	0	2	40	39
**Croatia**	2	2	0	0	5	87	64
**France**	3	2	0	1	11	221	150
**Germany**	3	3	0	0	10	200	176
**Hungary**	1	1	0	0	5	100	91
**Italy**	3	2	0	1	12	218	181
**Poland**	3	3	0	0	13	232	138
**Portugal**	1	0	0	1	1	20	20
**Romania**	1	1	0	0	3	60	56
**Slovenia**	1	1	0	0	4	80	65
**Spain**	2	1	0	1	12	145	115
**Ukraine**	1	1	0	0	5	100	92
**United Kingdom**	1	0	1	0	5	98	85
**Overall**	**25**	**18**	**2**	**5**	**98**	**1,788**	**1,425**

Table S2A. Overview of the number of different EP procedures per group as well as use of a 3D mapping system and/or contact force sensing catheters.

Procedure type	Overall number of procedures*	3D vs. no 3D*	CFSC vs. no CFSC*	3D + CFSC*	3D + no CFSC*	No 3D + CFSC*	No 3D + no CFSC*
**Group 1**	**646**	**237/409**	**120/526**	**104**	**133**	**16**	**393**
**AVNRT**	267	70/197	27/240	19	51	8	189
**Atrial flutter**	301	121/180	63/238	56	65	7	173
**RA AT**	38	27/11	20/18	20	7	0	11
**RA AP**	40	19/21	10/30	9	10	1	20
**Group 2**	**622**	**576/46**	**480/142**	**477**	**99**	**3**	**43**
**PVI (RF) w/o additional lines**	393	393/0	352/41	352	41	0	0
**PVI (RF) with additional lines**	122	122/0	95/27	95	27	0	0
**All PVIs (RF)**	515	515/0	447/68	447	68	0	0
**LA AT**	42	35/7	23/19	22	13	1	6
**LA AP**	65	26/39	10/55	8	18	2	37
**Group 3**	**157**	**148/9**	**125/32**	**125**	**23**	**0**	**9**
**PVC**	91	82/9	69/22	69	13	0	9
**VT**	66	66/0	56/10	56	10	0	0

Group 1: right-sided atrial EP procedures, group 2: left-sided atrial EP procedures, group 3: ventricular EP procedures. 3D = three-dimensional, CFSC = contact force sensing catheter, PVI = pulmonary vein isolation, RF = radiofrequency energy source, LA = left atrial, AT = atrial tachycardia, AP = accessory pathway, AVNRT = AV nodal reentrant tachycardia, RA = right atrial, PVC = premature ventricular contraction, VT = ventricular tachycardia. *Absolute numbers.

Table S2B. Fluoroscopy times with and without the use of contact force sensing ablation catheters for different EP procedures and according to the use of a 3D mapping system.

		Entire cohort			Procedures using 3D mapping systems only			Procedures w/o the use of 3D mapping systems only		
**Procedure type**	**Fluoro time†**	**Fluoro time with CFSC†**	**Fluoro time w/o CFSC†**	**p-value‡**	**Fluoro time with CFSC†**	**Fluoro time w/o CFSC†**	**p-value‡**	**Fluoro time with CFSC†**	**Fluoro time w/o CFSC†**	**p-value‡**
**Group 1**	**7.2 (2.6-15.0)**	**2.6 (0.0-6.0)**	**9.0 (4.0-16.0)**	**<0.001**	**2.0 (0.0-5.7)**	**2.0 (0.0-7.0)**	**0.473**	**5.8 (3.3-9.2)**	**11.0 (6.0-18.0)**	**<0.001**
**AVNRT**	8.0 (3.0-13.3)	2.6 (0.0-5.6)	8.8 (4.0-14.7)	<0.001	1.1 (0.0-3.7)	2.0 (0.0-7.0)	0.044	6.9 (4.5-10.3)	10.0 (6.0-16.0)	0.012
**Atrial flutter**	6.0 (2.0-15.0)	2.0 (0.0-6.0)	9.0 (3.2-16.0)	<0.001	2.0 (0.0-5.3)	2.0 (0.0-4.0)	0.624	4.6 (3.2-6.3)	11.0 (6.0-20.0)	0.064
**RA AT**	7.1 (4.0-20.0)	5.2 (1.0-16.0)	13.3 (6.0-22.0)	0.082	5.2 (1.0-16.0)	6.0 (0.6-8.0)	0.160	n/a*	22.0 (14.0-37.9)	n/a*
**RA AP**	8.5 (2.5-19.6)	1.0 (0.0-7.0)	10.5 (4.0-22.0)	<0.001	0.0 (0.0-3.0)	4.5 (0.0-8.0)	0.171	9.45**	17.6 (9.0-32.4)	n/a*
**Group 2**	**8.6 (4.6-16.8)**	**7.4 (4.0-13.2)**	**17.0 (7.0-38.0)**	**<0.001**	**7.4 (4.0-13.6)**	**16.4 (8.0-41.0)**	**<0.001**	**11.1 (5.0-12.0)**	**19.1 (6.2-25.7)**	**0.009**
**PVI (RF) w/o additional lines**	8.0 (4.2-14.0)	7.2 (4.0-13.0)	19.0 (11.0-33.7)	<0.001	7.2 (4.0-13.0)	19.0 (11.0-33.7)	<0.001	n/a*	n/a*	n/a*
**PVI (RF) with additional lines**	10.0 (5.0-20.0)	7.3 (5.0-14.0)	45.3 (34.9-61.8)	<0.001	7.3 (5.0-14.0)	45.3 (34.9-61.8)	<0.001	n/a*	n/a*	n/a*
**All PVIs (RF)**	8.4 (4.5-15.5)	7.2 (4.0-13.0)	27.4 (13.5-46.8)	<0.001	7.2 (4.0-13.0)	27.4 (13.5-46.8)	<0.001	n/a*	n/a*	n/a*
**LA AT**	9.0 (6.0-20.4)	8.5 (6.0-22.6)	10.0 (6.0-20.4)	0.293	8.3 (6.0-22.6)	7.0 (6.0-16.4)	0.586	12.0**	21.9 (11.2-27.7)	n/a*
**LA AP**	8.5 (4.0-21.0)	6.0 (4.0-11.1)	10.2 (4.0-21.7)	0.055	5.5 (2.2-12.5)	5.4 (0.5-8.0)	0.519	8.1 (5.0-11.1)	18.5 (6.2-24.8)	0.063
**Group 3**	**6.2 (2.5-14.0)**	**6.0 (2.2-12.7)**	**7.0 (3.0-20.3)**	**0.424**	**6.0 (2.2-12.7)**	**4.0 (2.5-20.0)**	**0.632**	**n/a***	**10.0 (7.5-20.6)**	**n/a***
**PVC**	5.0 (2.0-8.0)	4.0 (1.0-7.0)	7.8 (3.0-20.0)	0.016	4.0 (1.0-7.0)	5.2 (2.5-19.0)	0.193	n/a*	10.0 (7.5-20.6)	n/a*
**VT**	9.9 (4.0-23.0)	10.0 (5.0-23.0)	4.0 (3.0-35.6)	0.886	10.0 (5.0-23.0)	4.0 (3.0-35.6)	0.886	n/a*	n/a*	n/a*

Group 1: right-sided atrial EP procedures, group 2: left-sided atrial EP procedures, group 3: ventricular EP procedures. 3D = three-dimensional, CFSC = contact force sensing catheter, PVI = pulmonary vein isolation, RF = radiofrequency energy source, LA = left atrial, AT = atrial tachycardia, AP = accessory pathway, AVNRT = AV nodal reentrant tachycardia, RA = right atrial, PVC = premature ventricular contraction, VT = ventricular tachycardia. †Data are presented as medians with interquartile ranges. ‡Student's t-test with Welch modification. w/o = without, CFSC = contact force sensing catheter. *A group size of 0 observations (or only 1 observation) precludes calculation of a p-value.**

Table S2C. Procedure times with and without the use of contact force sensing ablation catheters for different EP procedures and according to the use of a 3D mapping system.

		Entire cohort			Procedures using 3D mapping systems only			Procedures w/o the use of 3D mapping systems only		
**Procedure type**	**Proced. time†**	**Proced. time with CFSC†**	**Proced. time w/o CFSC†**	**p-value‡**	**Proced. time with CFSC†**	**Proced. time w/o CFSC†**	**p-value‡**	**Proced. time with CFSC†**	**Proced. time w/o CFSC†**	**p-value‡**
**Group 1**	**78.5 (59.0-115.5)**	**101.0 (65.0-150.0)**	**75.0 (57.0-105.0)**	**<0.001**	**111.0 (70.0-155.0)**	**90.0 (60.0-118.0)**	**0.002**	**68.0 (57.0-81.0)**	**72.0 (56.0-96.0)**	**0.172**
**AVNRT**	78.0 (60.0-110.0)	80.0 (66.0-123.0)	76.0 (60.0-110.0)	0.253	106.0 (70.0-150.0)	95.0 (70.0-123.0)	0.936	72.5 (64.5-99.0)	74.5 (56.5-100.0)	0.697
**Atrial flutter**	70.0 (55.0-103.0)	98.0 (60.0-150.0)	70.0 (52.0-95.0)	<0.001	118.0 (65.0-160.0)	70.0 (45.0-103.0)	<0.001	59.0 (39.0-82.0)	70.0 (55.0-91.5)	0.139
**RA AT**	120.0 (90.0-140.0)	110.0 (71.0-138.0)	120.0 (93.0-170.0)	0.402	110.0 (71.0-138.0)	120.0 (100.0-160.0)	0.476	n/a*	120.0 (93.0-180.0)	n/a*
**RA AP**	90.0 (60.0-155.0)	162.5 (80.0-190.0)	86.5 (40.0-120.0)	0.025	165.0 (150.0-190.0)	105.0 (70.0-130.0)	0.154	80.0**	81.5 (38.5-120.0)	n/a*
**Group 2**	**128.0 (92.0-177.0)**	**130.0 (93.5-170.0)**	**124.0 (85.0-180.0)**	**0.721**	**130.0 (94.0-170.0)**	**135.0 (90.0-200.0)**	**0.377**	**120.0 (25.0-123.0)**	**110.0 (70.0-130.0)**	**0.528**
**PVI (RF) w/o additional lines**	124.0 (92.0-167.0)	123.0 (90.0-162.5)	135.0 (100.0-180.0)	0.122	123.0 (90.0-162.5)	135.0 (100.0-180.0)	0.122	n/a*	n/a*	n/a*
**PVI (RF) with additional lines**	169.5 (130.0-210.0)	153.0 (127.0-200.0)	210.0 (180.0-240.0)	0.001	153.0 (127.0-200.0)	210.0 (180.0-240.0)	0.001	n/a*	n/a*	n/a*
**All PVIs (RF)**	135.0 (97.0-180.0)	130.0 (95.0-172.0)	180.0 (115.0-210.0)	<0.001	130.0 (95.0-172.0)	180.0 (115.0-210.0)	<0.001	n/a*	n/a*	n/a*
**LA AT**	114.5 (60.0-180.0)	117.0 (70.0-215.0)	110.0 (36.0-177.0)	0.306	117.5 (75.0-215.0)	40.0 (36.0-177.0)	0.243	25.0**	125.0 (70.0-150.0)	n/a*
**LA AP**	94.5 (65.0-121.5)	107.5 (70.0-123.0)	92.0 (65.0-120.0)	0.978	102.5 (63.0-130.0)	70.0 (60.0-90.0)	0.418	121.5 (120.0-123.0)	108.5 (75.0-127.5)	0.227
**Group 3**	**130.0 (92.0-261.5)**	**144.0 (102.5-232.0)**	**100.0 (80.0-127.5)**	**<0.001**	**144.0 (102.5-232.0)**	**115.0 (80.0-140.0)**	**0.012**	**n/a***	**100.0 (80.0-110.0)**	**n/a***
**PVC**	110.5 (80.0-159.0)	120.0 (83.5-180.0)	90.0 (75.0-115.0)	<0.001	120.0 (83.5-180.0)	90.0 (75.0-115.0)	0.001	n/a*	100.0 (80.0-110.0)	n/a*
**VT**	180.0 (123.0-260.0)	182.5 (130.0-271.5)	135.0 (120.0-225.0)	0.199	182.5 (130.0-271.5)	135.0 (120.0-225.0)	0.199	n/a*	n/a*	n/a*

Group 1: right-sided atrial EP procedures, group 2: left-sided atrial EP procedures, group 3: ventricular EP procedures. Proced. = procedure, 3D = three-dimensional, CFSC = contact force sensing catheter, PVI = pulmonary vein isolation, RF = radiofrequency energy source, LA = left atrial, AT = atrial tachycardia, AP = accessory pathway, AVNRT = AV nodal reentrant tachycardia, RA = right

atrial, PVC = premature ventricular contraction, VT = ventricular tachycardia. †Data are presented as medians with interquartile ranges. ‡Student's t-test with Welch modification. w/o = without, CFSC = contact force sensing catheter. *A group size of 0 observations (or only 1 observation) precludes calculation of a p-value.**
